# Recent Update Roles of Magnetic Nanoparticles in Circulating Tumor Cell (CTC)/Non-CTC Separation

**DOI:** 10.3390/pharmaceutics15102482

**Published:** 2023-10-17

**Authors:** Chawapon Pipatwatcharadate, Poornima Ramesh Iyer, Dakrong Pissuwan

**Affiliations:** 1 Nanobiotechnology and Nanobiomaterials Research (N-BMR) Laboratory, School of Materials Science and Innovation, Faculty of Science, Mahidol University, Bangkok 10400, Thailand; pipat_cp@hotmail.com (C.P.); poornima.iye@student.mahidol.edu (P.R.I.); 2 Materials Science and Engineering Program, Faculty of Science, Mahidol University, Bangkok 10400, Thailand; 3 Center of Excellence on Medical Biotechnology (CEMB), Faculty of Science, Mahidol University, Bangkok 10400, Thailand

**Keywords:** magnetic nanoparticles, circulating tumor cells, separation, gold nanoparticles, antibody

## Abstract

Metastasis of cancer is a major cause of death worldwide. Circulating tumor cells (CTCs) are important in the metastatic process of cancer. CTCs are able to circulate in the bloodstream. Therefore, they can be used as biomarkers of metastasis. However, CTCs are rare when compared to a large number of blood cells in the blood. Many CTC detection methods have been developed to increase CTC detection efficiency. Magnetic nanoparticles (MNPs) have attracted immense attention owing to their potential medical applications. They are particularly appealing as a tool for cell separation. Because of their unique properties, MNPs are of considerable interest for the enrichment of CTCs through CTC or non-CTC separation. Herein, we review recent developments in the application of MNPs to separate CTCs or non-CTCs in samples containing CTCs. This review provides information on new approaches that can be used to detect CTCs in blood samples. The combination of MNPs with other particles for magnetic-based cell separation for CTC detection is discussed. Furthermore, different approaches for synthesizing MNPs are included in this review.

## 1. Introduction

Cancer is a leading cause of death worldwide. The International Agency for Research on Cancer has recently reported that the estimated number of cancer-related deaths from 2020 to 2025 is over 10 million. The primary cause of death in cancer patients is metastasis, which is a complex process [1]. Some cells migrate away from the primary tumor and spread through the blood stream and/or lymphatic vessels to colonize cancer cells in specific new tissues during metastasis [2]. Circulating tumor cells (CTCs) were discovered by an Australian pathologist, Thomas Ashworth, in 1869 during autopsy in metastasized cancer patients [3]. These cells are also called tumor-derived epithelial cells. They initiate cancer metastasis even in the early stages of tumorigenesis [4]. However, cancer patients have a few CTCs in peripheral blood and lymph capillaries [5]. CTCs distributed in blood are aggressive cells that can escape recognition by immune cells and shear stress of bloodstream [6,7]. It has been reported that approximately one CTC corresponds to 10^9^ hematologic cells from metastatic cancer patients [8]. CTCs are promising for use in cancer diagnosis and treatment because they can act as multifunctional biomarkers and biological signals of cancer disease progression. Furthermore, CTCs can be detected by a noninvasive liquid biopsy in clinical diagnosis [9,10]. These CTCs can provide vital information related to cancer, especially disease progression and metastasis. An expression of genetic materials can also help provide information about the primary tumor [11].

As previously mentioned, the number of CTCs in the bloodstream is generally very low and they are mixed with many white and red blood cells (RBCs) [12]. Hence, their detection and separation are difficult. In 1950–1959, CTC separation was performed by density gradient centrifugation [13,14]. However, this approach has a high cost and is inconvenient. Therefore, several technologies have been developed to detect and isolate CTCs, e.g., filtration [15], microfluidic [16], and density-based cell separation [17]. Extensive research is being performed to increase the efficacy of CTC detection and isolation. Because of their unique properties, nanomaterials are increasingly being used in a lot of diagnostic applications [18,19], such as bacterial [20] and adhesion molecule [21] detection. Nanomaterials have also been used in detecting and analyzing CTCs owing to their ability for efficient cellular binding and friendly fabrication [22]. The magnetic nanoparticles (MNPs) used in medicine are mainly iron-based and they have received considerable attention for detecting or separating target cells. They demonstrate a high potential for cell separation; for example, cancer cells are separated from fresh whole blood using MNPs conjugated with antibodies against human epithelial growth factor receptor 2 [23]. The use of MNPs to separate a protein called cluster of differentiate 20 (CD20)-positive lymphoma cells has also been reported [24]. Therefore, they play an important role in cell separation, including CTC separation through magnetic separation process. It is worth noting that the size of MNPs used for cell separation has a wide range at the nanoscale level [25]. It has been reported that capture efficiency can rely on the aggregate size scale of MNPs, MNP concentration, and magnetic field intensity used for separation [26].

In this review, we demonstrate the recent progress in CTC detection using MNPs to capture CTCs or separate non-CTC cells from blood samples. The review begins by providing information on the synthesis of MNPs followed by the use of MNPs for CTC detection. The combination of MNPs with other metal nanoparticles for CTC detection are also included. Finally, the perspective of using MNPs in CTC research is discussed in detail.

## 2. Synthesis of MNPs through Chemical Approach

**Figure 1:**
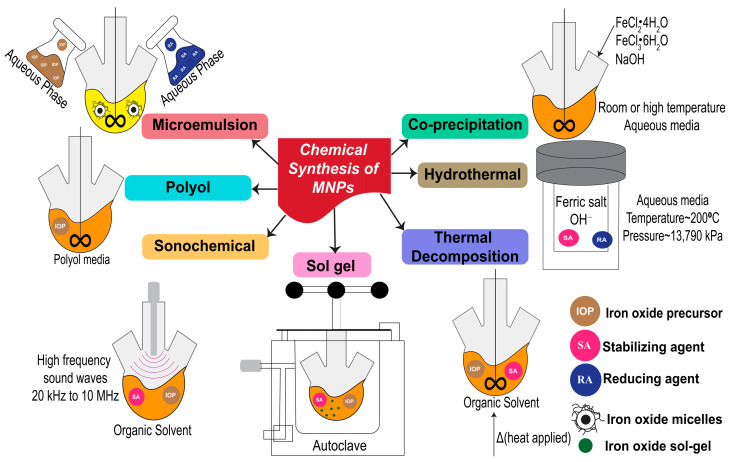
Schematic illustration of primary chemical methods used for synthesizing MNPs.

Much effort has been invested to develop potential methods for MNP synthesis. However, the properties of MNPs can impact their utilization. For example, MNPs with remarkable stability and the ability to switch to a magnetic state are required for data storage applications. In the case of biomedical application, nanoparticles with superparamagnetic properties are attractive. In addition, MNPs that are stable in water at neutral pH or in a physiological environment are suitable for diagnostics and therapeutics [25]. According to a recent review by Shukla et al. [27], MNPs can be prepared via three methods. The first one is a physical method with a top–down approach. The other two methods are based on chemical and biological mechanisms with a bottom–up approach. This review discusses only the chemical method, as it is simple and can produce uniform-sized MNPs. Various chemical methods have been used to synthesize MNPs, the most important of which are discussed in the following subsections. A schematic illustration of the primary chemical methods is provided in Figure 1.

### 2.1. Co-Precipitation

Co-precipitation is the famous method for MNP synthesis due to its simplicity and high yield [28]. Furthermore, this method is relatively easy, with better control over the size and property of MNPs [29]. The co-precipitation process involves the use of ferrous and ferric iron in an aqueous salt solution, followed by adding a base at room or high temperature. The size of MNPs obtained by this method can vary from 5 to 40 nm depending on the type of salts, pH of the alkaline solution, and reaction time [30,31]. The surfaces of MNPs synthesized by co-precipitation can also be easily modified with biocompatible polymers [32]. The requirement for surface modification of MNPs is not only to create non-toxic and biocompatible surfaces; but, also, to enhance specific target binding by attaching specific ligands to target cells on the surface of MNPs. The size of MNPs synthesized by this technique depends on the salt type, ferric and ferrous ion ratio, pH value, and temperature during synthesis [33]. MNPs synthesized using this method are widely used in biomedical applications because of their favorable properties.

### 2.2. Thermal Decomposition

Thermal decomposition is suitable for producing uniform-sized MNPs. Ansari et al. [34] discussed two techniques used for thermal decomposition. The first method involves heating a mixture of metal precursors (such as iron carbonyls), surfactants, and organic solvents to induce the clustering and growth of MNPs [35,36]. The second technique involves injecting reagents into a heated surfactant solution. Small particle size ranges of 3–20 nm MNPs can be obtained using high temperatures (such as ~265 °C) [33]. This technique can stimulate a rapid and homogeneous nucleation. Common surfactants used in this technique are oleates and acetylacetonates. Benzyl ether or octadecene is regularly used as an organic solvent [37] for this purpose.

### 2.3. Hydrothermal Method

MNPs can be synthesized through a hydrothermal method using an autoclave at a high pressure and temperature. Iron precursors such as sulfates and chlorides can be used in this method [38]. Natarajan et al. [39] reported that the temperature and pressure used for this method were higher than 200 °C and 13,790 kPa, respectively. However, the synthesis of MNPs using a lower temperature of 140 °C for 6 h has also been reported. This indicates that heating time also affects the process in this method [40]. It has been reported that temperature, pH, and precursor type affect the size of MNPs. For example, at 130 °C, MNPs with sizes of 16–40 nm were obtained when iron sulfate was used as a precursor. However, smaller sizes of 5–20 nm were formed using iron chloride as a precursor [38]. Furthermore, hydrothermal conditions in the presence and absence of surfactants can also affect the size and magnetic property of MNPs. This method is attractive for preparing MNPs with varying sizes and shapes [41].

### 2.4. Polyol Method

In addition to using high temperatures, the polyol method is an alternative way to synthesize MNPs at a low-temperature range (~25 °C). High temperatures can be used depending on the boiling points of the solvents used in this method. Iron salts are dissolved in a polyol solution such as polyethylene glycol (PEG). This technique is famous for preparing metal nanoparticles with well-defined shapes and sizes (ranging from 10–150 nm) [42]. A mixture of ferrous hydroxide in organic media and polyols can provide stable MNPs because polyols can act as stabilizers of MNPs [39]. It is important to note that the type and concentration of polyol impact the size, shape, and yield of MNPs.

### 2.5. Microemulsion

Another technique that can be used to synthesize MNPs is the microemulsion method. This technique uses two immiscible phases of water and oil to form a stable colloidal suspension after the two phases coincide to form a single phase [27]. Some reports have demonstrated that the MNPs produced via this technique can have a small size with high magnetization [41,43]. The surfactant used in this procedure strongly affects the properties of the resultant MNPs [44,45,46]. Therefore, the surfactant must be chosen carefully to obtain the desired properties of MNPs. To obtain small sized MNPs at ~4 nm, sodium 2-ethylhexyl sulfosuccinate was used as a surfactant with heptane in the oil phase [46].

### 2.6. Sonochemical Method

The sonochemical method, in combination with ultrasound, is an easy technique for nanomaterial synthesis. Tiny acoustic bubbles stimulated by sound waves can produce energy, and these bubbles can efficiently host chemical reactions. MNPs can be effectively synthesized using the sonochemical method. This method involves acoustic cavitation via bubble formation and growth, which collapses in a liquid medium. The primary sonochemistry starts with localized hot spot generation after rapid bubble collapse at transient temperatures up to approximately 230 °C and cooling rates over 10^3^ K s^−1^. Oxidant radicals, the secondary sonochemistry, are stimulated after the primary sonochemistry to reach stable phases and allow for salt conversion into MNPs [47,48]. Suslick et al. [49] synthesized MNPs using this method. Iron pentacarbonyl and decane were used as the precursor and the solvent, respectively. A surfactant (such as polyvinylpyrrolidone and oleic acid) was used to stabilize the surface of MNPs and size ranges of 3–8 nm were obtained.

### 2.7. Sol-Gel Method

The sol-gel method uses hydroxylation and condensation to form nanoparticles. The part of forming solid nanoparticles are generally called a part of the sol. After that, condensation and polymerization are applied to remove the solvent or chemical reactions are performed with metal oxide (in this case, iron oxide) to form a network of three-dimensional metal oxides. This network is a part of the wet gel, which is later treated with heat to obtain a crystalline structure [27]. The use of ferric nitrate with citric acid or ethylene glycol as precursors for the synthesis of MNPs used in this method has been previously reported [33,50]. Similar to other methods, temperature, pH, and precursors are the major parameters affecting the physical properties of the MNPs.

After the synthesis, the morphology and size of the MNPs can be characterized using electron microscopy. The zeta potential values of MNPs, as well as their stability, as indicated by the polydispersity index (PDI), can be measured using dynamic light scattering. The functional groups on the surface of MNPs can be investigated using Fourier transform infrared spectroscopy (FTIR). The net magnetization of MNPs can be measured using a superconducting quantum interference device or vibrating sample magnetometry [34].

## 3. CTC/Non-CTC Separation by MNPs

The MNPs used in cell separation play a vital role in diagnosis. Therefore, they have recently received considerable attention as tools for cell separation in samples containing more than one type of cell [24,51]. As mentioned earlier, CTCs are extremely rare in the bloodstream, which also contains other cell types. A lot of reports have demonstrated the role of MNPs in rare cell diagnoses, especially in CTCs test samples. Therefore, here we discussed how MNPs can be used to separate CTCs (target detecting cells) or other non-CTCs (target non-detecting cells or non-CTCs).

### 3.1. CTC Separation by MNPs

**Figure 2:**
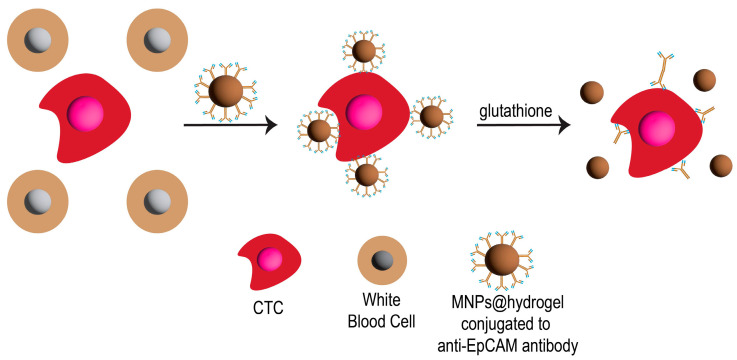
Schematic illustration of using MNPs coated with a hydrogel conjugated to anti-EpCAM for the CTC separation and recovery after applying a magnetic scaffold.

Separating CTCs from test samples can be performed by directly targeting them with MNPs conjugated to specific targeting moieties (e.g., antibodies) to CTCs. For example, Wang et al. [52] investigated the use of MNPs to capture CTCs by fabricating MNPs with a hydrogel layer that could inhibit nonspecific cell adhesion. The hydrogel was composed of zwitterionic poly(sulfobetaine methacrylate) (pSBMA) and methacrylic acid (MAA) cross-linked with *N*,*N*-bis(acryloyl)cystamine (BACy). The hydrogel layer was covalently conjugated to an anti-epithelial cell adhesion molecule (anti-EpCAM) antibody through the carboxyl group of MAA (Figure 2). This antibody could attach to their model CTCs (high-expressing EpCAM cells; MCF-7 cells) resulting in having a high potential for CTC separation from test samples after applying with a magnet scaffold. A solution of glutathione (GSH) was used to break the disulfide bonds in the hydrogel matrix to separate the MNPs. This could then separate MNPs from CTCs and achieve more than 95% of CTC viability.

**Figure 3:**
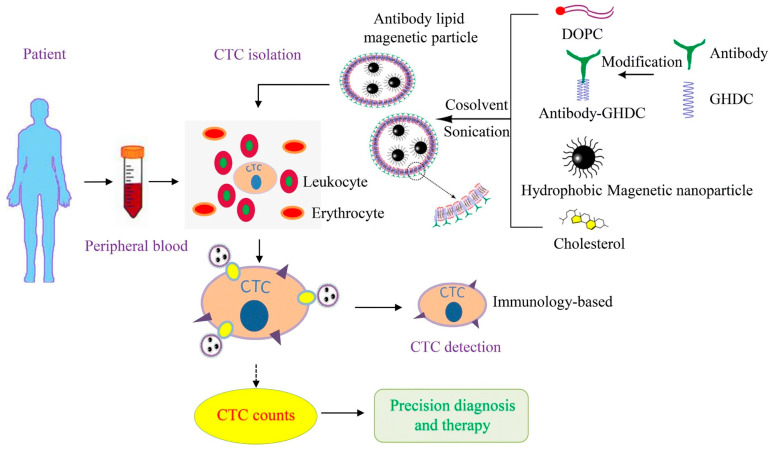
Schematic illustration of lipid MNPs for CTC separation [53]. Copyright 2019, The Author(s), Springer Nature.

Aside from EpCAM antibodies, other antibodies used for targeting CTCs in combination with MNPs have also been reported. Chen et al. [53] reported the single use of magnetic immunoliposomes (lipid MNPs) conjugated to antibody or combined use of each type of lipid MNPs conjugated to antibody on CTC separation. Four different antibodies (EpCAM, epidermal growth factor receptor (EGFR), human epidermal growth factor receptor-2 (HER-2), and Mucin-1 (MUC-1)) were used for conjugation with lipid MNPs. The performance of CTC separation in blood samples by single or combined use was investigated. (Figure 3). The lipid layer reduced the instability of MNPs in blood samples. This could improve the efficiency of CTC separation. When comparing the single (non-mixing of each antibody conjugated to lipid MNPs) and combined (mixing of EpCAM@lipid MNPs, EGFR@lipid MNPs, HER-2@lipid MNPs, and MUC-1@lipid MNPs) use, the single use had a higher efficiency in CTC separation than that of the combined use. This information is useful for designing MNPs for CTC separation.

Another example has been demonstrated by Haghighi et al. [54]. A conjugation between MNPs and herceptin was prepared. Herceptin is an antibody that can selectively target HER-2 receptors, which are overexpressed on breast, ovarian, lung, and gastric cancers at approximately 20–30%. The covalent binding occurred through amine groups (-NH_2_) functionalized on the surface of MNPs and carboxyl groups on herceptin. The conjugated MNPs were used to separate human breast cancer SKBR3 cells from the blood. After applying a magnetic field, SKBR3 cells were separated from the blood samples using a low amount of herceptin conjugated to MNPs. This technique has a potential economic cost for CTC separation using MNPs.

Although antibody-conjugated MNPs have a high affinity and selectivity to target antigens, they also have certain disadvantages [55]. Antibodies are large molecules with molecular weights of ~150 kDa. This large size limits the number of antibodies conjugated to MNPs. In addition, the random and non-directional orientation of the conjugated antibodies can reduce immunoreaction efficiency. Moreover, antibody-based techniques are limited by their stability, reproducibility, large-scale production, high cost, and accuracy. For example, a false-negative result caused by non-specific expression of EpCAM on normal epithelial cells was reported [56]. This can reduce the efficiency of CTC detection. Owing to these disadvantages, some researchers have focused on conjugating peptides instead of antibodies to MNPs to target CTCs. The large difference in molecular weight between the peptide (~2.7 kDa) and the antibody (~150 kDa) resulted in the surface of MNPs obtaining higher numbers of peptide molecules than antibody molecules [57]. Furthermore, the binding affinity of peptides to target ligands is similar to or greater than that of the antibodies. In addition, peptides provide higher stability and reproducibility than antibodies, and can be mass-produced via chemical synthesis [58].

A recent work by Liang et al. [57] used peptide-functionalized MNPs (Pep@MNPs) to separate CTCs from blood samples. The peptide had a binding affinity for lung cancer cells (A959 and NCI-H1975 cells). The percentage of captured CTCs was similar to that obtained using the anti-EpCAM antibodies. Using this peptide, they also found that it reduced the false-negative rate of CTC detection in blood samples of early stage non-small cell lung cancer (NSCLC) patients. In a similar study conducted by Jia et al. [59], a novel peptide targeting N-cadherin was conjugated to MNPs. This peptide targeted N-cadherin, which is overexpressed in mesenchymal CTCs (transforming growth factor β (TGF-β)-induced MCF-7 cells). Their novel peptide conjugation could separate mesenchymal CTCs) in spiked blood samples containing mesenchymal CTCs at ~85% capture efficiency.

**Figure 4:**
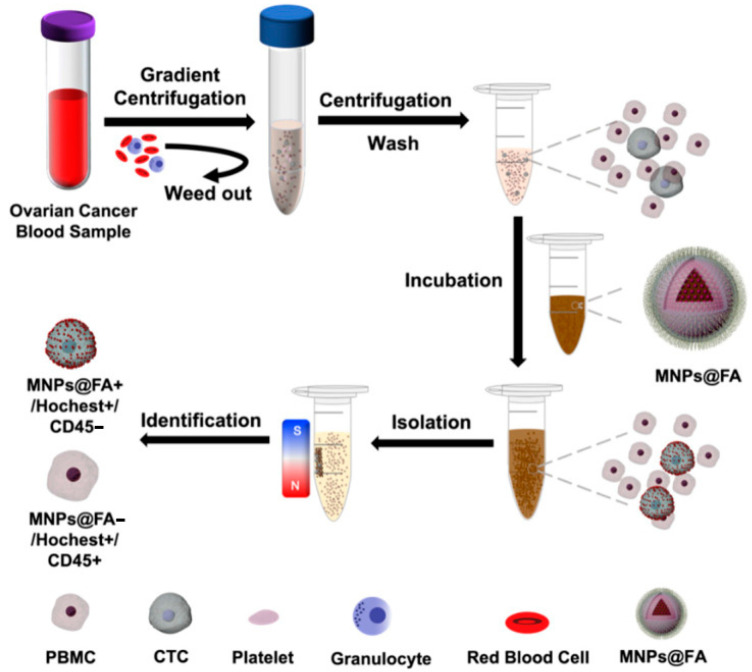
Schematic illustration of CTC separation using FA@MNPs [60]. Copyright 2022, The Author(s), Multidisciplinary Digital Publishing Institute.

Small molecular probes, such as folic acid (FA), are of particular interest to researchers. Similar to other peptides, FA has a lower molecular weight than antibodies. This acid contains a few functional groups that allow for better orientation, is inexpensive, and has low immunogenicity. Furthermore, FA has a strong affinity binding to folate receptors expressed on various types of cancer cells. A recent work by Pan et al. [60] demonstrated the use of surface-modified MNPs conjugated to FA (FA@MNPs) to separate CTCs from blood samples of patients with ovarian cancer. The CTCs separated using FA@MNPs had a high rate of viable cells, which could be further used for biological investigation of ovarian cancer information in vitro. The process of using FA@MNPs for CTC separation is illustrated in Figure 4.

The use of cystamine-mediated FA conjugated to MNPs for CTC separation was reported by Li et al. [61]. PEG was used as a linker to link FA to cystamine-modified MNPs. After CTCs (HeLa cells) were spiked in lysed blood, a high percentage of CTC capture (≥80% depending on the number of CTCs spiked in blood) occurred. Healthy CTCs could be released from the particles after treating with dithiothreitol to break the disulfide bonds of cystamine. These released CTCs were useful for gaining more information of cancer through proteomic analyses. Another FA-functionalized MNP was demonstrated by Meng et al. [62]. The FA molecules were conjugated to a poly(amidoamine) dendrimer (PAMAM-FA) via a linker (PEG) and then further attached to biotin to form biotin-PAMAM-FA. After MNPs were conjugated with streptavidin (SA) to form SA-MNPs, they could bind to biotin-PAMAM-FA, resulting in the separation of CTCs (ovarian cancer cells; SKOV3 cells) with high separation efficiency and cell viability of ~90%.

**Figure 5:**
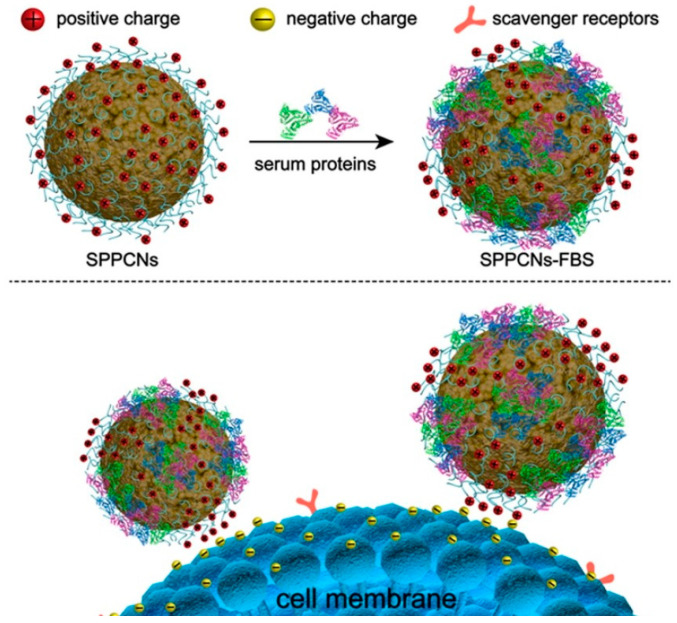
Schematic illustration of CTC separation without labelling target ligands specific to CTCs on MNPs [63]. Copyright 2020, American Chemical Society.

Wu et al. [63] demonstrated a label free technique for CTC separation by poly(ethyleneimine)-functionalized MNPs (Figure 5). This technique relies on an electrostatic interaction between functionalized MNPs and target CTCs. This interaction is based on serum protein-coating on the surface of MNPs, which later could attach to epithelial protein expression in different types of cancer cells. The efficiency of the interaction between cancer cells and MNPs also depended on the incubation time of the cells and MNPs, as well as the medium component. The CTC separation efficiency of spiked CTC in blood samples 47–72%, 68%, and 78–89% regarding to the number of cancer cells at 10, 100, and 1000 cells spiked in healthy whole blood at a volume of 1 mL. In this technique, blood is also lysed to remove RBCs. Notably, there was no binding competition between CTCs and white blood cells (WBCs) for MNP attachment.

Apart from the aforementioned work, another interesting approach has been developed to improve the efficiency for capturing CTCs using MNPs. A biomimetic system was used in cooperation with MNPs. The membrane originating from RBC vesicles was used to coat the surface of MNPs. This membrane can help to avoid the adsorption of nonspecific biomolecules in biological fluids. The targeted antibodies were then attached to the coated MNPs. This technique could increase the CTC separation efficiency in spiked blood samples with PC-3 cells by ~35% compared with using MNPs attached to targeted antibodies without RBC membranes. This technique provides good potential for CTC separation by MNPs [64]. In addition to the RBC membrane, neutrophil membranes can be used for similar proposes by coating on the surface of MNPs. This membrane can reduce nonspecific protein adsorption and WBC interruption during CTC capture resulting in an enhanced separation efficiency of CTCs by MNPs [65]. It can be seen that the bioconjugation of MNPs with molecules that can target CTCs has a vital role in CTC separation. The use of label free MNPs and biomimetic MNPs has also attracted increasing attention in CTC targeting. These biofunctionally designed MNPs have different advantages and disadvantages as shown in Table 1.

**Figure 6:**
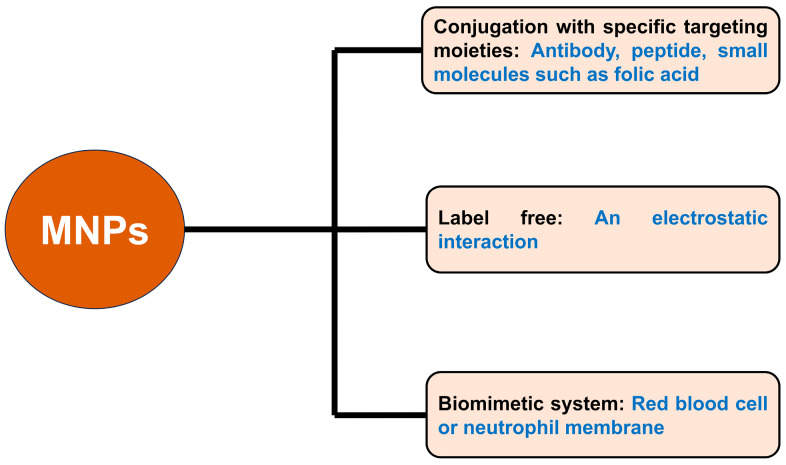
Overview of different approaches of using MNPs to capture CTCs.

An overview approach of using MNPs to capture CTCs is shown in Figure 6. As mentioned above, there are some disadvantages in targeting or capturing CTCs. For example, distinguishing CTC isolation from other cell populations in blood samples has a low efficiency. Furthermore, an extra process to separate CTCs from MNPs for further downstream analysis is needed. To overcome these disadvantages, the separation of non-CTCs from test samples can be carried out. This approach is discussed in the next section.

### 3.2. Separation of Non-CTCs by MNPs

**Figure 7:**
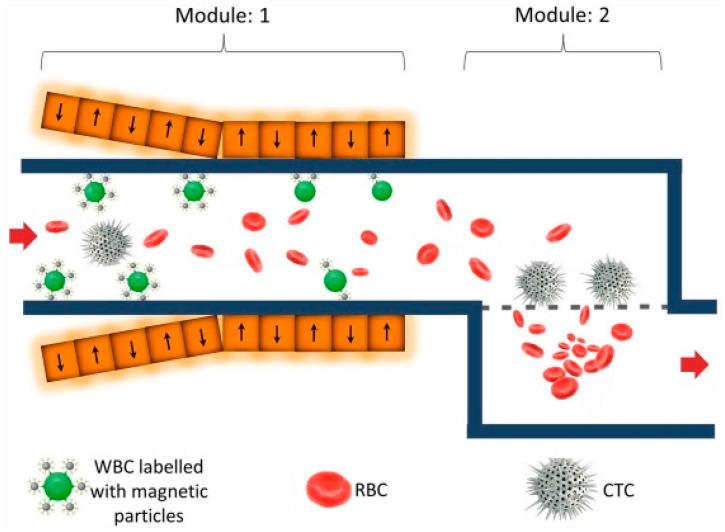
Schematics of a chip platform with two microfluidic modules. The first module uses MNPs to remove WBCs and the second module uses a size-based separation to remove the RBCs and retain CTCs. The orange squares with arrows represent the permanent magnetic arrays [66]. Copyright 2016, Elsevier.

It is well-known that there are many types of cells in blood samples. The separation of these non-CTCs cells from the test sample can help to enrich the number of CTCs in the test sample. As previously discussed, MNPs can be used to capture CTCs. A similar principle can be used to capture different cell types. In this section, we focus on the use of MNPs to capture non-CTCs. The first technique created a microfluidic chip that involved 2 major processes to enrich CTCs after depletion of non-CTCs. First, MNPs were labelled with a specific antibody that targets CD54 antigens expressed on WBCs. The second stage involved the removal of RBCs and platelets via size-based separation. Finally, >80% of the CTCs were recovered from 2 mL of spiked blood samples [66]. A schematic representation of this technique is presented in Figure 7.

The use of MNPs in an integrated microfluidic chip to isolate WBCs from blood samples was also reported by Lee et al. CTCs [67]. MNPs were conjugated with the anti-CD45 antibody, resulting in reduced interference from WBCs. This device can increase the efficiency of sorting CTCs in blood samples. Similar to the previous technique mentioned above, MNPs can also potentially deplete non-CTCs from blood samples. Although microfluidic technologies are preferred for separating CTCs from whole blood, they still require complex design and manufacturing.

**Figure 8:**
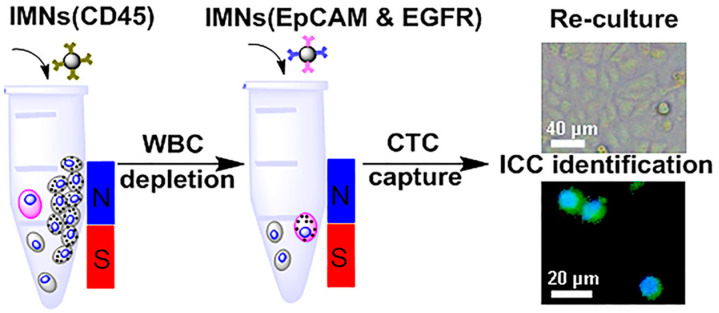
Schematic illustration of anti-CD45-conjugated to MNPs (IMNs(CD45)) and anti-EpCAM and anti-EGFR antibodies conjugated to MNPs (IMNs(EpCAM and EGFR)) for isolating WBCs (white drawing cells in the tube) and capturing CTCs (a pink drawing cell in the tube). Cocktails of anti-EpCAM and anti-EGFR antibodies can effectively capture tumor cells and then immunocytochemistry (ICC) identification was performed. The anti-CD45 antibody can target and isolate non-CTCs (WBCs) after magnetic-field attachment (a N-S drawing rod attached to the tube) [68]. Copyright 2018, American Chemical Society.

Ma et al. [68] also developed MNPs conjugated with anti-CD45 antibody to remove WBCs from lysed RBCs of blood samples. Thereafter, cocktails of anti-EpCAM and anti-EGFR antibodies were conjugated to the MNPs and used to capture CTCs. By using two antibodies instead of one, the recognition of CTCs became more efficient, as a result of being able to better target CTCs after a large number of WBCs was depleted (Figure 8). Compared with microfluidic chips, this technique is simple and can provide good efficiency for CTC enrichment and purification.

Overall, the use of MNPs to separate non-CTCs showed that MNPs are useful for removing non-CTCs from the test sample. This approach helps increase the number of CTCs. Furthermore, the use of MNPs to target CTCs after non-CTC separation also increases CTC purity.

## 4. CTC Detection by Combining MNPs with Other Materials

As discussed in the previous section, the combination of non-CTC and CTC capture can increase the efficiency of CTC purification using MNPs. However, there are two major disadvantages to using MNPs for cell separations. First, the formation of MNP clusters around cells after application of a magnetic field can cause difficulties in targeted cell quantification. The second one is the damage to the targeted cell by MNPs after a magnetic field is applied [69]. In this case, the optimized properties of the MNPs, such as size, morphology, surface modification, and magnetic field applied for separation, are considered. Recent reports suggest that MNPs can be combined with other materials to improve CTC detection. For example, the recent work by Chang et al. [70] demonstrated a hybrid structure of MNPs and gold nanoparticles (GNPs) for CTC isolation and detection. The leukocyte membranes were used to coat the proposed immunomagnetic GNPs to obtain leukocyte membrane-camouflaged immunomagnetic GNPs. These particles were further developed by conjugation with the EpCAM antibodies. Chang and the team found that immunomagnetic GNPs had a high specificity for EpCAM-positive CTCs. Additionally, the interaction between leukocytes and immunomagnetic GNPs decreased due to the leukocyte membrane on the immunomagnetic GNPs. After the immunomagnetic GNPs were applied to healthy human blood samples spiked with 100–5000 MCF-7 breast cancer cells, the CTCs were rapidly detected. This technique can be further developed to gain a high signal that can be used for cancer progress detection.

**Figure 9:**
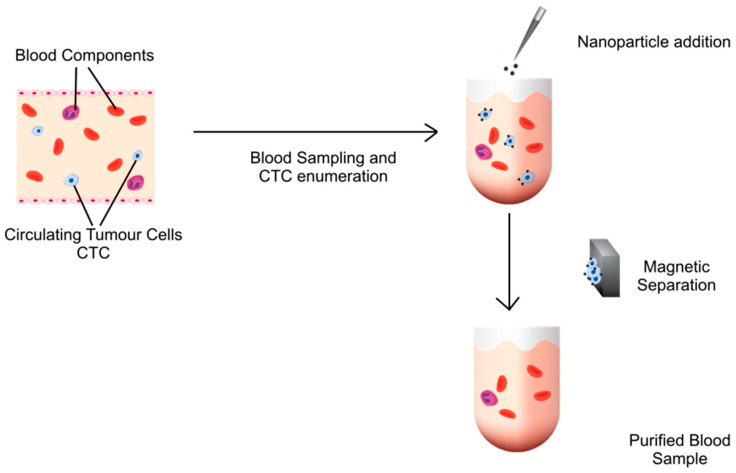
Schematic showing the CTC isolation from a blood sample using magnetic carbon-coated cobalt nanoparticles EpCAM antibodies [71]. Copyright 2022, The author(s), Multidisciplinary Digital Publishing Institute.

Another recent work using MNPs and EpCAM antibodies was reported by Doswald et al. [71]. They proposed using magnetic carbon-coated cobalt nanoparticles conjugated with EpCAM antibodies to isolate CTCs from the blood samples of metastatic carcinoma patients. With their designed particles, the CTCs could be isolated (~≥68%) without interference from other blood components (Figure 9).

**Figure 10:**
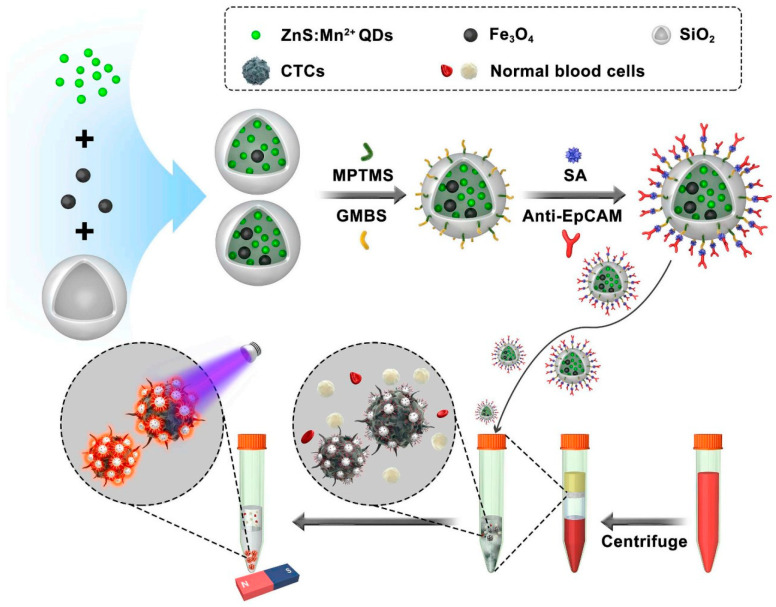
Schematic showing the isolation of CTCs from a blood sample of breast cancer patients using immune-nanocomposite nanoparticles conjugated with anti-EpCAM antibodies [72]. Copyright 2019, Elsevier.

Cui et al. [72] demonstrated another novel isolation approach using immunonanocomposites of ZnS:Mn^2+^ quantum dots and MNPs encapsulated in SiO_2_ nanospheres and then conjugated to anti-EpCAM antibodies (Figure 10). A rapid solation process and high binding efficiency of CTCs to nanocomposites were found after applying these immunonanocomposites to the patients’ blood samples who had breast cancer. Because of ZnS:Mn^2+^ quantum dots, these immunonanocomposite nanoparticles provided a CTC-specific yellow-orange fluorescent signal with 90.8% capture efficiency.

## 5. Conclusions

This review clearly shows that MNPs have a great potential for CTC/non-CTC separation, which is helpful for CTC detection. The use of MNPs in both separations can improve CTC detection. The design of biofunctional molecules on the surface of MNPs before use, to capture target CTCs/non-CTCs, is important to achieve cell separation using MNPs. As shown in Table 1, the non-specific binding of MNPs can strongly affect the separation efficiency of CTCs including non-CTCs. Therefore, it is necessary to design MNPs with a high efficiency for CTC capture. Further development of biofunctionalized MNPs is required to improve the outcome of CTC capture, separation, and detection. Removing non-CTCs from samples using MNPs can enrich CTCs. Nevertheless, it is important to consider how to design biofunctionalized MNPs with high affinity and specificity for target cells. Notably, the performance of MNPs for CTC/non-CTC separation relies on three vital factors: (1) the specificity of the ligands conjugated the MNP surface to the molecules expressed on target CTCs/non-CTCs; (2) the separation mechanism designed to isolate labeled cells; and (3) the facility used for CTC detection after separation. The combination of other materials with MNPs can also help enhance the favorable properties of MNPs that facilitate the capture CTCs with a high detection signal.

## 6. Future Perspective

With the promising outcomes of using MNPs for CTC/non-CTC separation, MNPs can be a crucial tool for rapid detection of CTCs in the blood samples of cancer patients by depleting non-CTCs and enriching CTCs. Further development of the specially designed MNPs to enhance the accuracy and efficiency of CTC detection in blood samples of patients with cancer should be addressed in future studies. However, it is important to explore whether MNPs can be potentially used to capture CTCs in the clinical blood samples with a high accuracy. Separating viable CTCs from clinical samples is also important to obtain informative cancer data. To achieve this, a simple approach with a highly efficient capture of CTCs is required. In addition, a magnetic field platform for cell separation using a comfortable strategy should be considered. Calibration and evaluation using MNPs and other established approaches are required for both CTC/non-CTC separation. Furthermore, MNPs are scalable and easy to synthesize. This can enhance the MNPs ready for CTC/Non-CTC separation of large clinical volumes. Overall, MNPs can be useful for precise cell separation, which can help in the initial extensive screening and monitoring of the disease progression in patients with cancer.

## Figures and Tables

**Table 1:** Advantages and disadvantages of biofunctionally designed MNPs for CTC separation.

Designed MNPs	Advantages	Disadvantages
Antibody-conjugated MNPs	▪ High affinity and selectivity to target antigens	▪ Limitation of antibody molecules on the surface of MNPs owing to the high molecular weight of antibodies ▪ Reduction in immunoreaction efficiency because of random and non-directional orientation of the conjugated antibodies ▪ Low stability and poor reproducibility ▪ Uncertain accuracy and high cost ▪ Limitation in large-scale production
Small biomolecule (such as peptide, folic acid) -conjugated MNPs	▪ High numbers of molecules on on the surface of MNPs owing to their small molecular weights ▪ High affinity and selectivity to target antigens ▪ High stability and reducibility ▪ Mass production via chemical synthesis ▪ Having good orientation	▪ High cost
Biomimetic MNPs	▪ High separation efficiency in spiked blood samples ▪ Reduction nonspecific protein adsorption and white blood cell	▪ Requirement for bioconjugation (with antibodies or small biomolecules) on the surface of biomimetic MNPs ▪ High cost
Label free MNPs	▪ Lower cost than biofunctionally designed MNPs ▪ Short processing time	▪ Low affinity and selectivity to target CTCs
